# Supplementary figures and images for: Differential bicodon usage in lowly and highly abundant proteins
Source: PeerJ. 2017 Mar 9;5:e3081. doi: 10.7717/peerj.3081 (PMC5346287; doi:10.7717/peerj.3081)

# of sequences

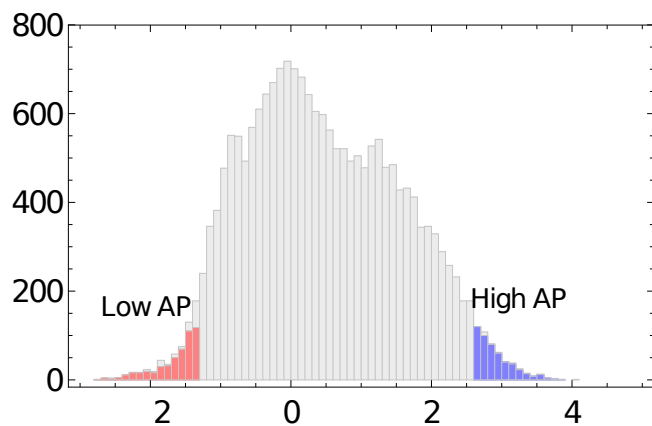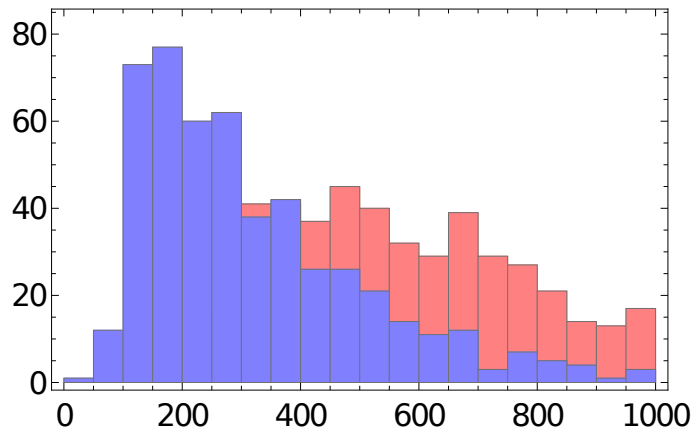

# of sequences

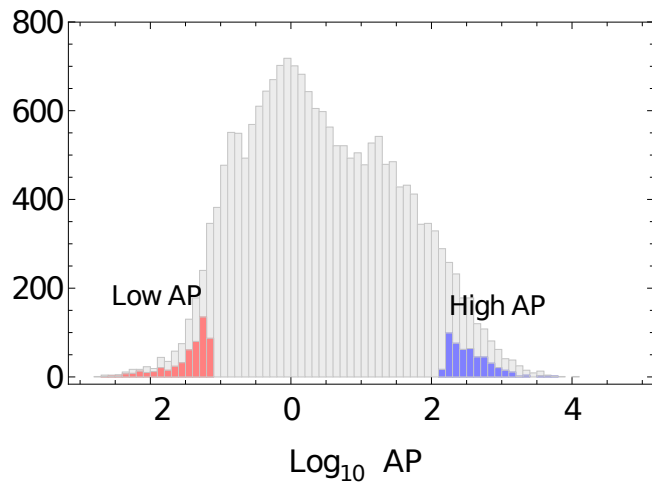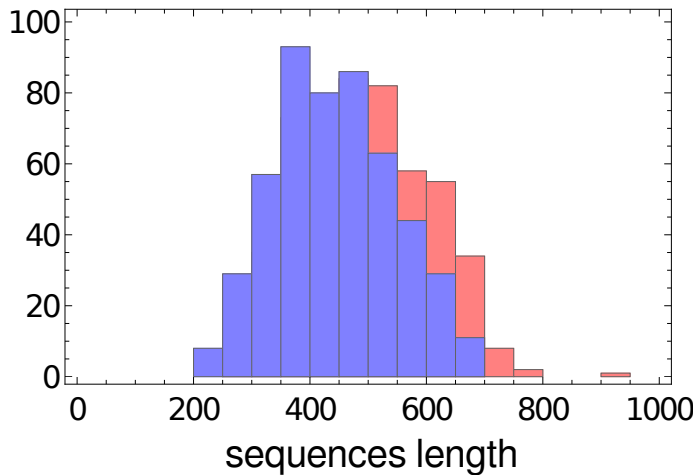

Supplement: Supplemental Information 4 — The protein abundance distributions of the whole dataset, lowest and highest protein abundance subsets are indicated in red and blue colors, respectively (A). The sequence length distributions of the subsets of sequences are shown in the left panel (B). The protein abundance distributions of the whole dataset, and the selected low and high protein abundance subsets of sequences used in the study are indicated in red and blue colors, respectively (C). The sequence length distributions corresponding to the subsets of sequences are shown in the left panel (D). [file peerj-05-3081-s004.pdf]

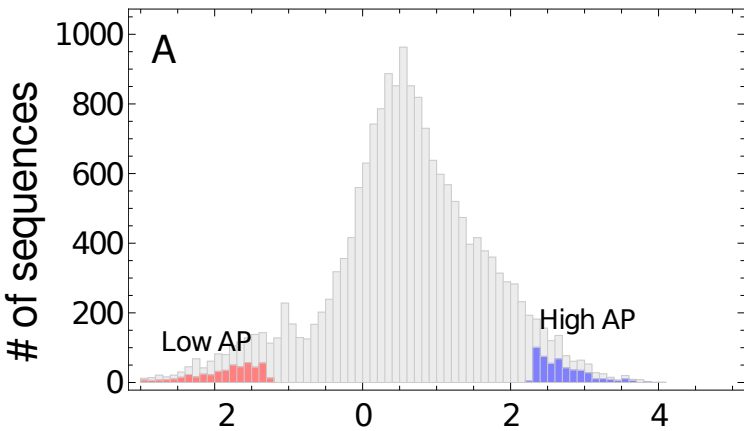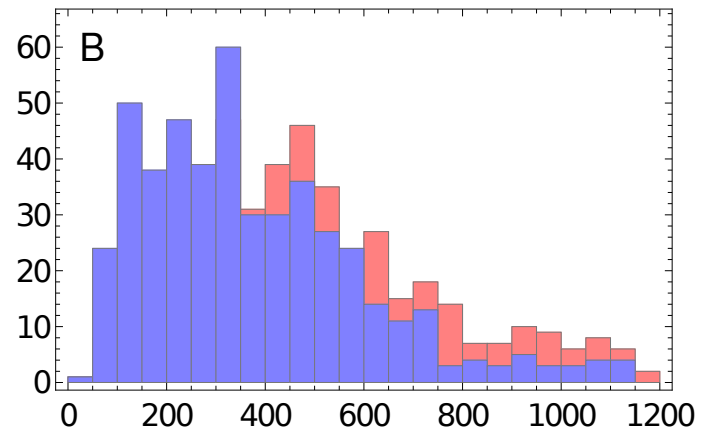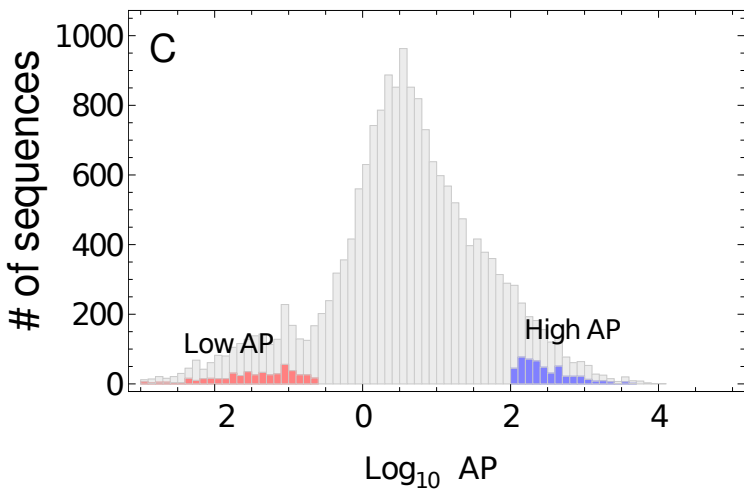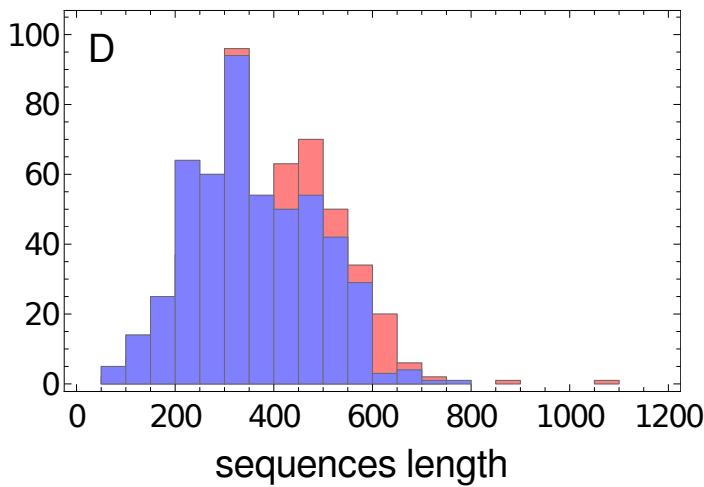

Supplement: Supplemental Information 5 — The protein abundance distributions of the whole dataset, lowest and highest protein abundance subsets are indicated in red and blue colors, respectively (A). The sequence length distributions of the subsets of sequences are shown in the left panel (B). The protein abundance distributions of the whole dataset, and the selected low and high protein abundance subsets of sequences used in the study are indicated in red and blue colors, respectively (C). The sequence length distributions corresponding to the subsets of sequences are shown in the left panel (D). [file peerj-05-3081-s005.pdf]

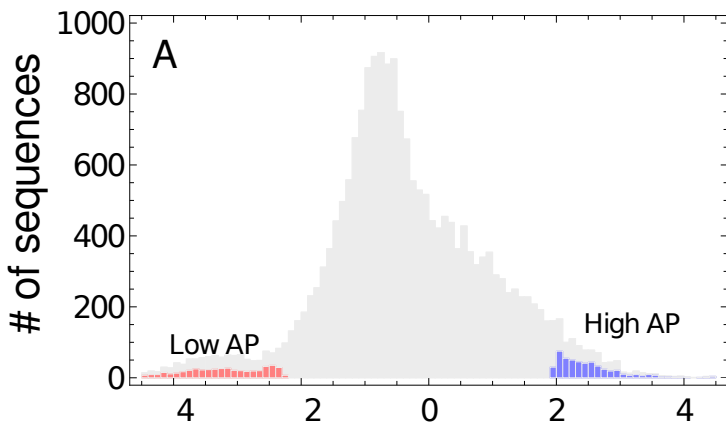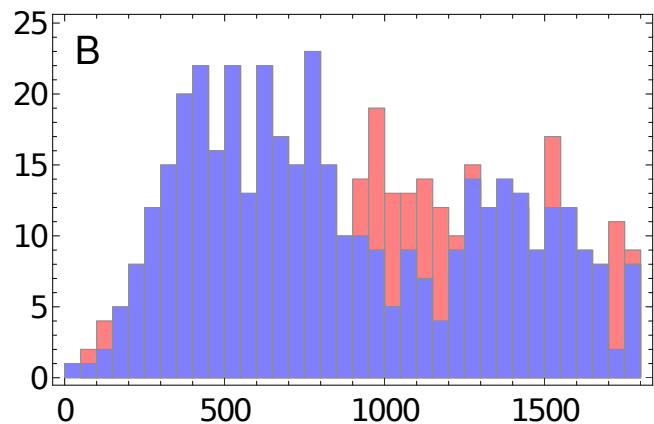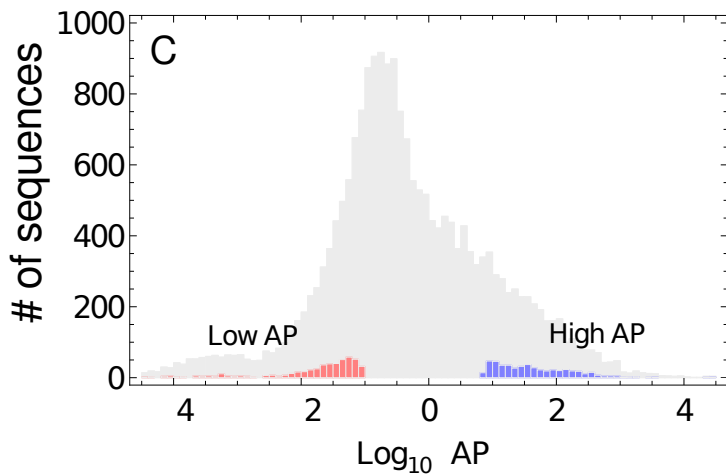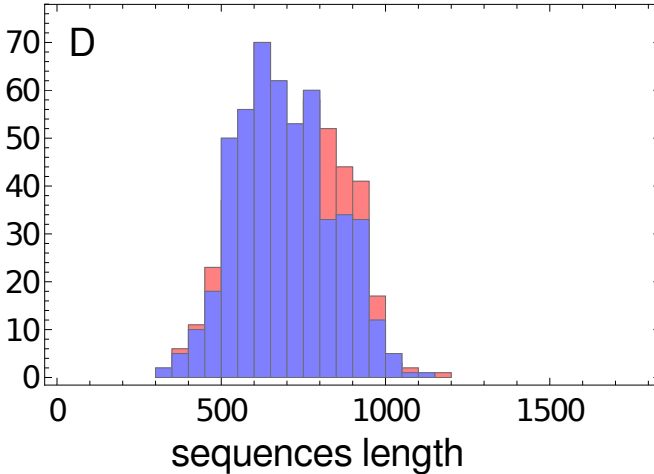

Supplement: Supplemental Information 6 — The protein abundance distributions of the whole dataset, lowest and highest protein abundance subsets are indicated in red and blue colors, respectively (A). The sequence length distributions of the subsets of sequences are shown in the left panel (B). The protein abundance distributions of the whole dataset, and the selected low and high protein abundance subsets of sequences used in the study are indicated in red and blue colors, respectively (C). The sequence length distributions corresponding to the subsets of sequences are shown in the left panel (D). [file peerj-05-3081-s006.pdf]

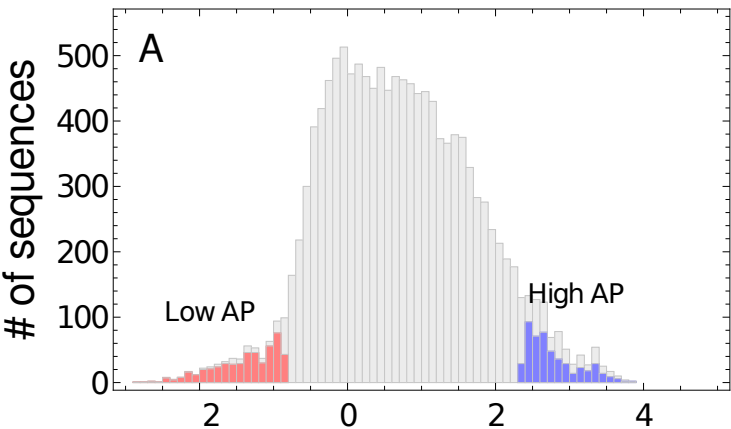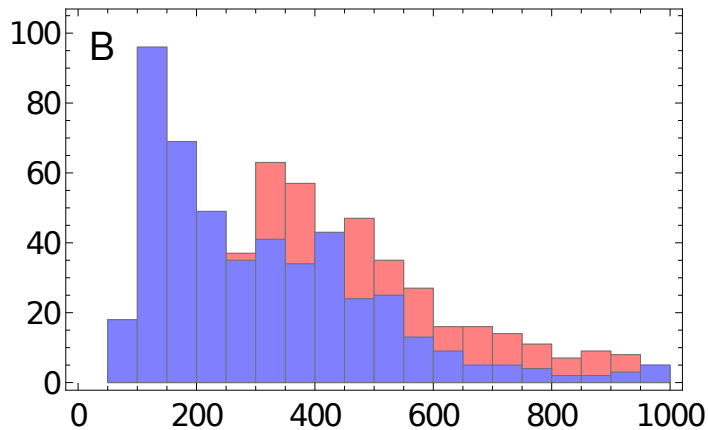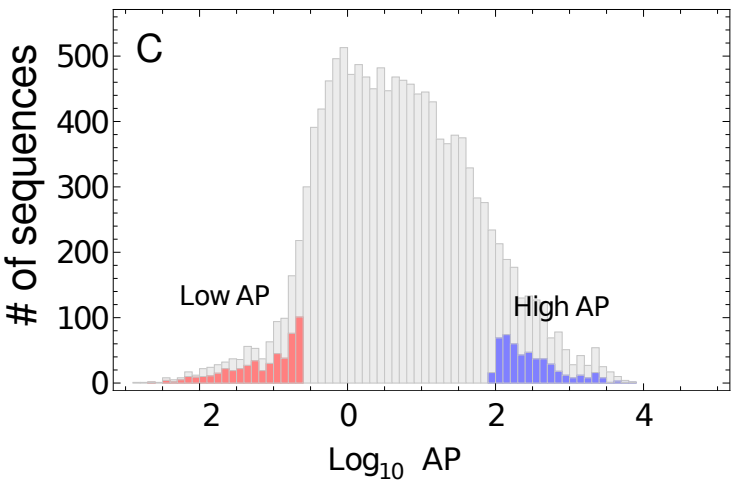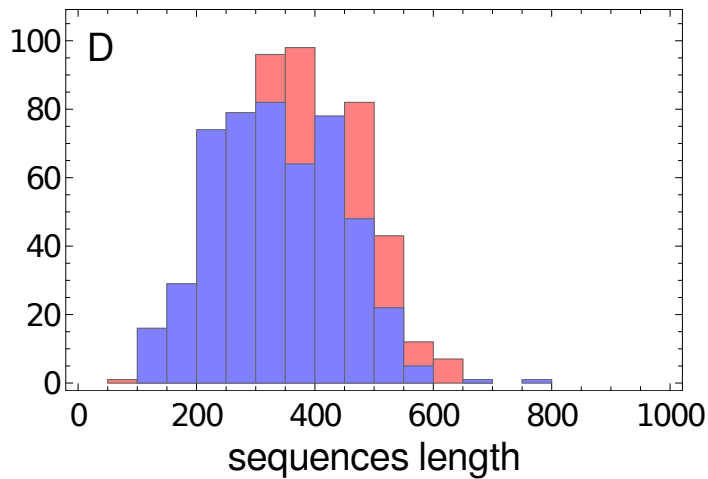

Supplement: Supplemental Information 7 — The protein abundance distributions of the whole dataset, lowest and highest protein abundance subsets are indicated in red and blue colors, respectively (A). The sequence length distributions of the subsets of sequences are shown in the left panel (B). The protein abundance distributions of the whole dataset, and the selected low and high protein abundance subsets of sequences used in the study are indicated in red and blue colors, respectively (C). The sequence length distributions corresponding to the subsets of sequences are shown in the left panel (D). [file peerj-05-3081-s007.pdf]

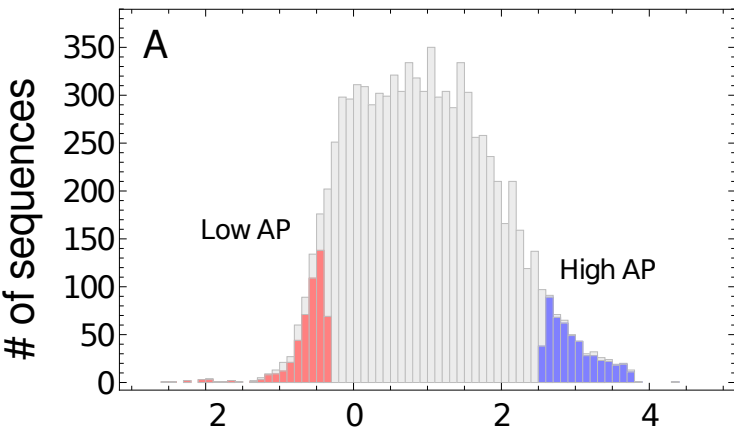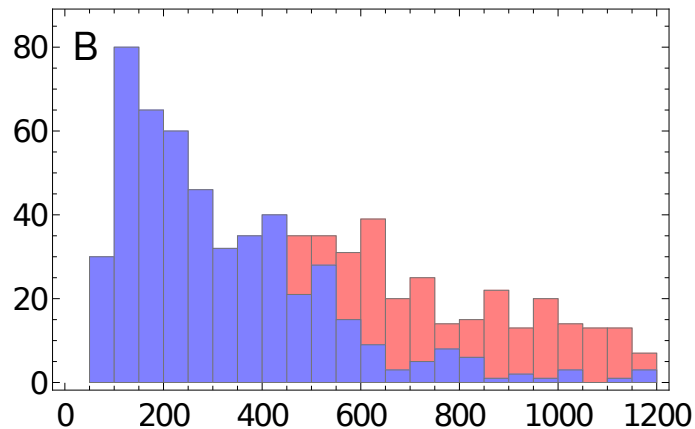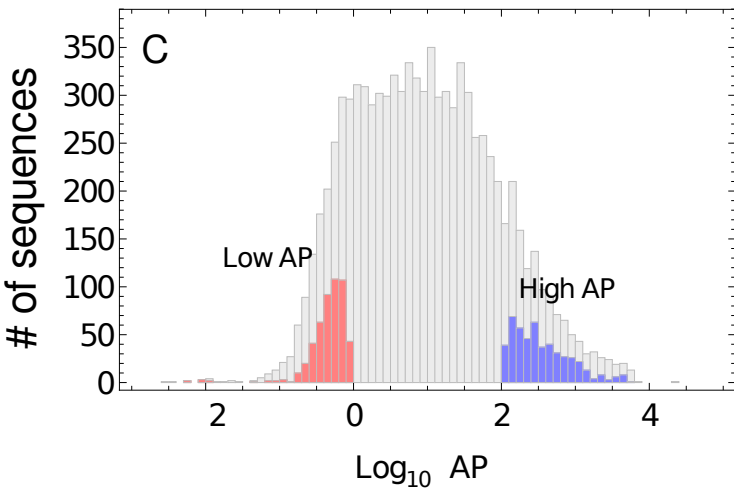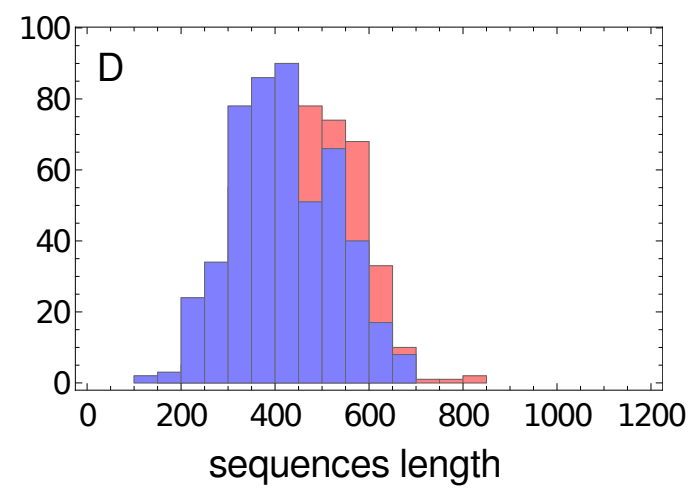

Supplement: Supplemental Information 8 — The protein abundance distributions of the whole dataset, lowest and highest protein abundance subsets are indicated in red and blue colors, respectively (A). The sequence length distributions of the subsets of sequences are shown in the left panel (B). The protein abundance distributions of the whole dataset, and the selected low and high protein abundance subsets of sequences used in the study are indicated in red and blue colors, respectively (C). The sequence length distributions corresponding to the subsets of sequences are shown in the left panel (D). [file peerj-05-3081-s008.pdf]

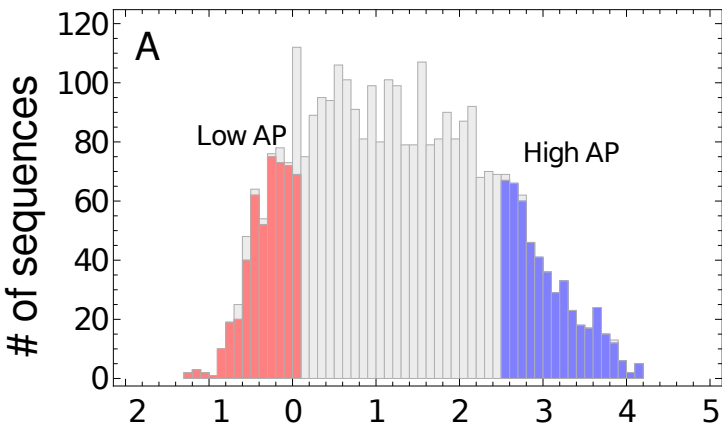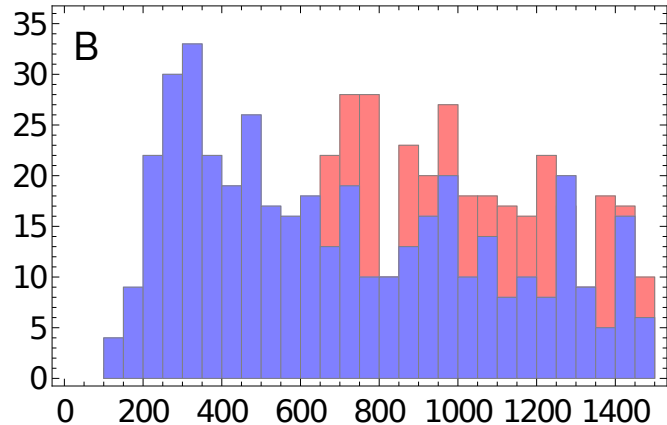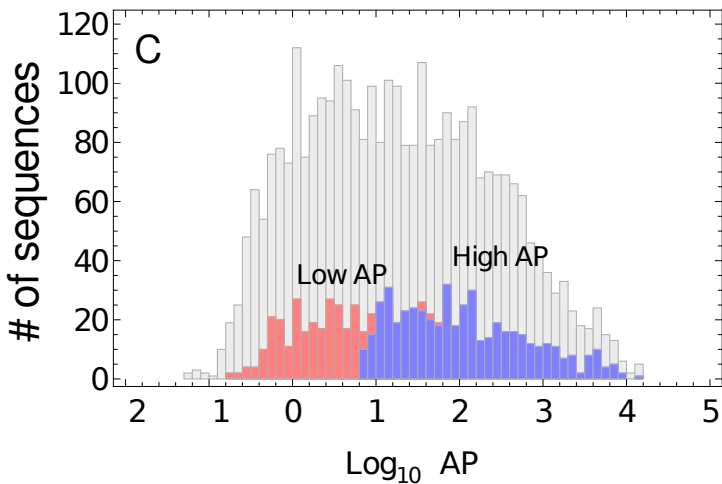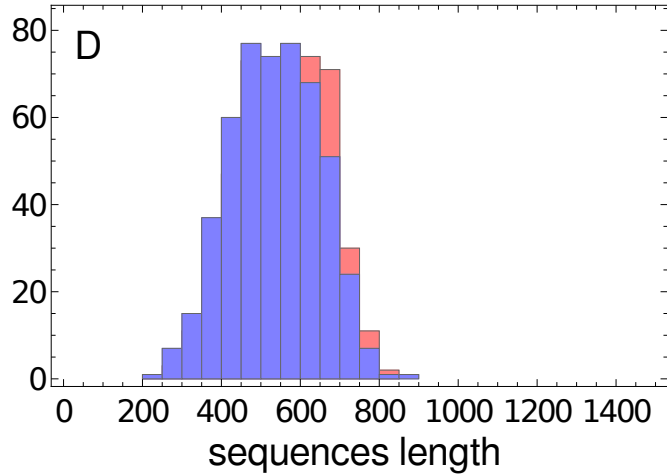

Supplement: Supplemental Information 9 — The protein abundance distributions of the whole dataset, lowest and highest protein abundance subsets are indicated in red and blue colors, respectively (A). The sequence length distributions of the subsets of sequences are shown in the left panel (B). The protein abundance distributions of the whole dataset, and the selected low and high protein abundance subsets of sequences used in the study are indicated in red and blue colors, respectively (C). The sequence length distributions corresponding to the subsets of sequences are shown in the left panel (D). [file peerj-05-3081-s009.pdf]

# of sequences

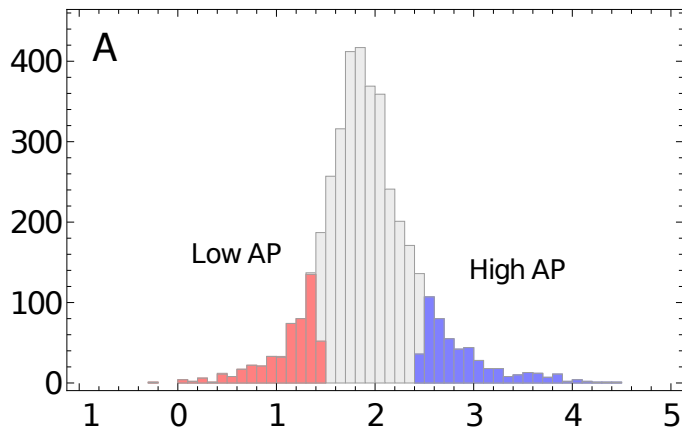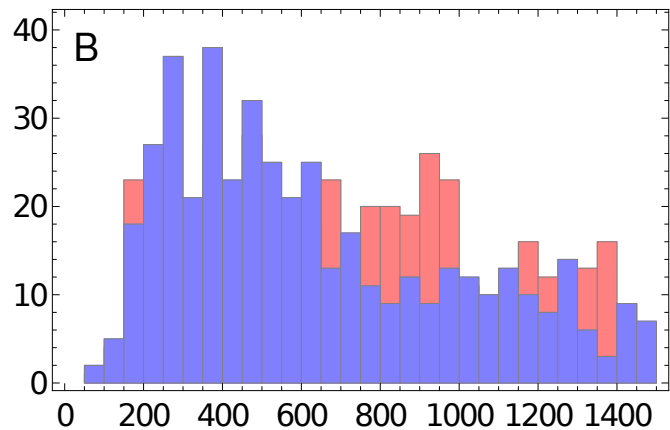

# of sequences

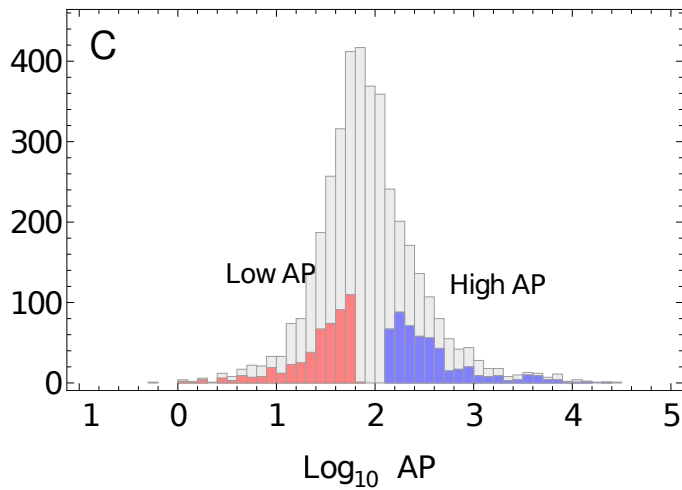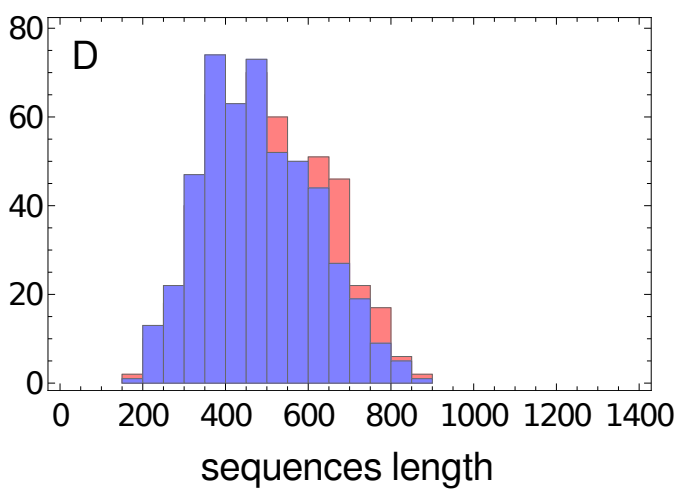

Supplement: Supplemental Information 10 — The protein abundance distributions of the whole dataset, lowest and highest protein abundance subsets are indicated in red and blue colors, respectively (A). The sequence length distributions of the subsets of sequences are shown in the left panel (B). The protein abundance distributions of the whole dataset, and the selected low and high protein abundance subsets of sequences used in the study are indicated in red and blue colors, respectively (C). The sequence length distributions corresponding to the subsets of sequences are shown in the left panel (D). [file peerj-05-3081-s010.pdf]

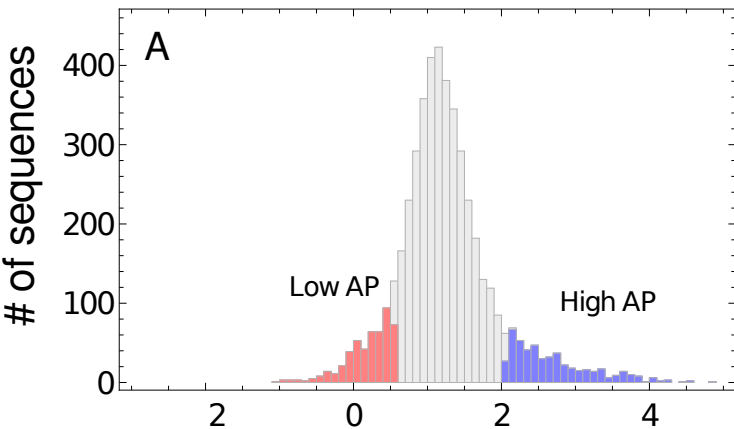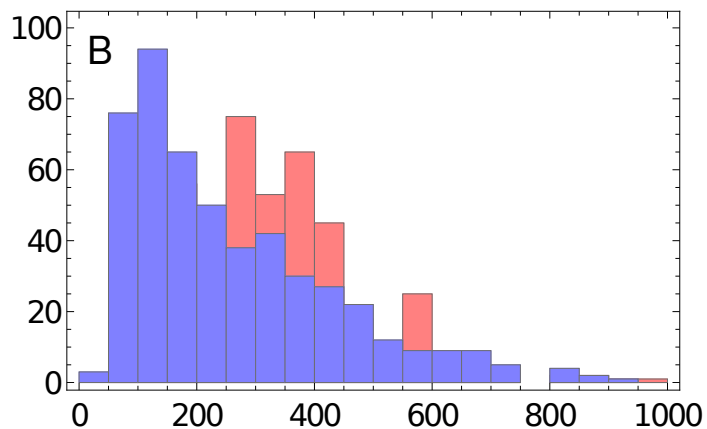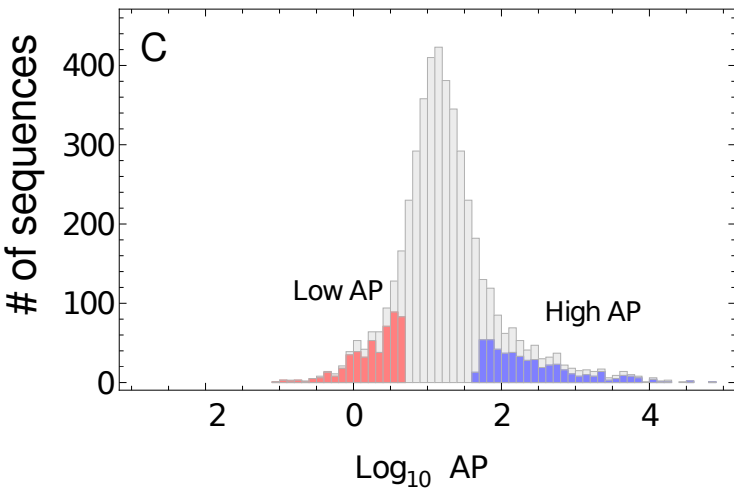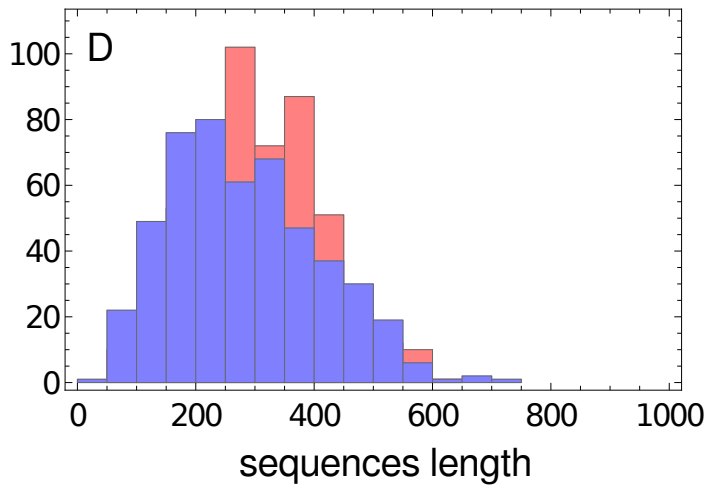

Supplement: Supplemental Information 11 — The protein abundance distributions of the whole dataset, lowest and highest protein abundance subsets are indicated in red and blue colors, respectively (A). The sequence length distributions of the subsets of sequences are shown in the left panel (B). The protein abundance distributions of the whole dataset, and the selected low and high protein abundance subsets of sequences used in the study are indicated in red and blue colors, respectively (C). The sequence length distributions corresponding to the subsets of sequences are shown in the left panel (D). [file peerj-05-3081-s011.pdf]

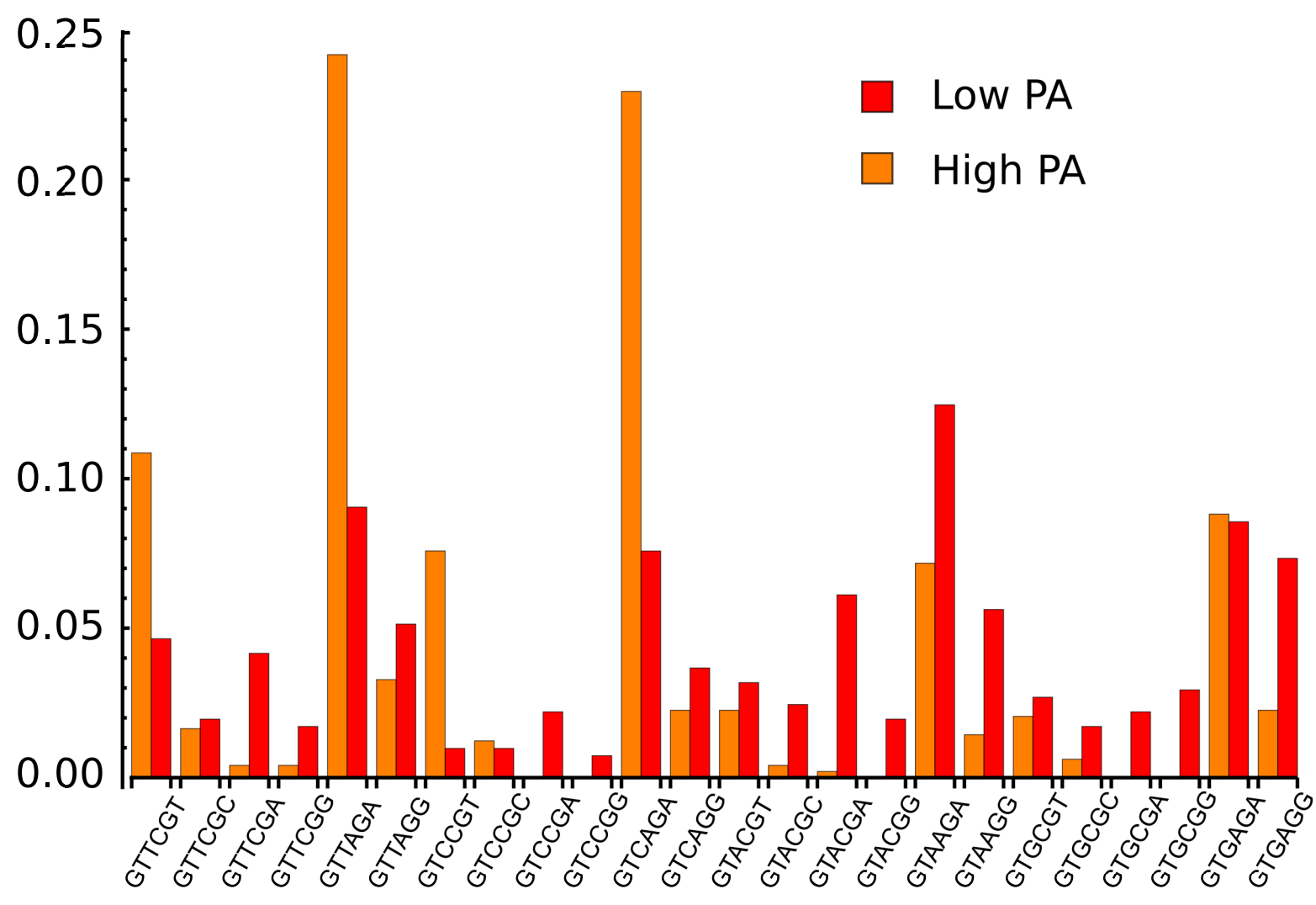

Supplement: Supplemental Information 13 — Frequencies associated with bicodons that encode the amino acid pair VR, computed using sequences from low PA sample (red bars) and from high PA sample (orange bars). The frequency usage of bicodon GTGAGA in the sequences of both samples are almost the same, while other bicodons have an evident preference for sequences associated with low or high PA. [file peerj-05-3081-s013.pdf]

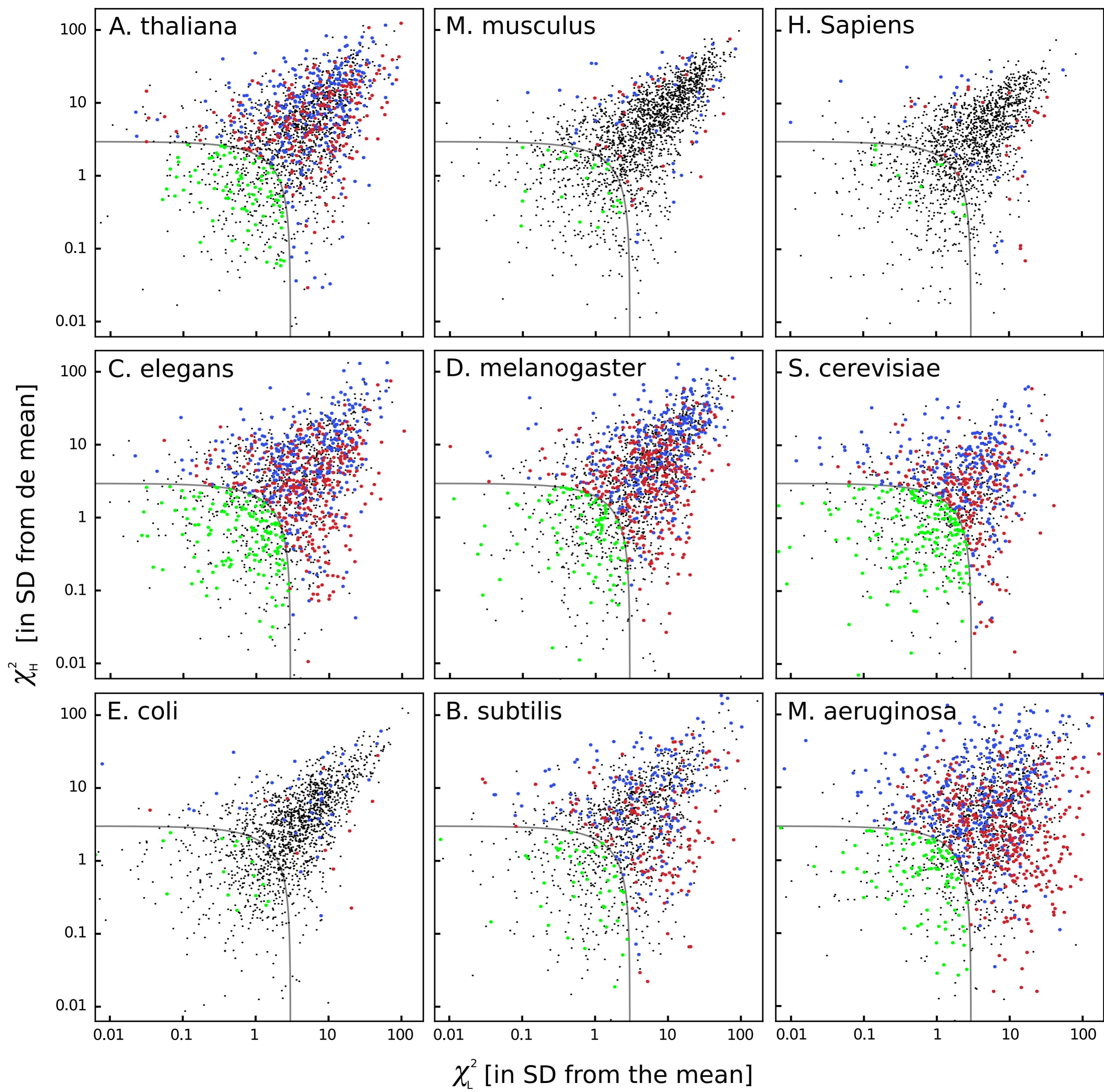

Supplement: Supplemental Information 16 — Scatter plots indicating the residual scores χL and χH computed over low PA and high PA samples, respectively. The codon pairs whose preference for sequences with low or high PA cannot be explained for codon usage bias are outside the grey quadrant (i.e., χ2 > 3 × SD). Among them, we distinguish bicodons more frequently used in low PA sequences (red dots), or in high PA sequences (blue dots). Inside the quadrant, there are codon pairs with a significantly different usage frequency in low and high PA samples, but such bias can be explained for codon usage bias (green dots). Codon pairs whose usage frequencies in low and high PA samples are not significantly different (black dots). [file peerj-05-3081-s016.pdf]
