# Supplementary material for: Differential bicodon usage in lowly and highly abundant proteins
Source: PeerJ. 2017 Mar 9;5:e3081. doi: 10.7717/peerj.3081 (PMC5346287; doi:10.7717/peerj.3081)
Supplement: Supplemental Information 12 — In the left table we show the observed occurrence for the 4 bicodons that encode the amino acid pair KK in low and high PA sequences samples of S. cerevisiae. From this table we can obtain one 2 × 2 contingency table for each bicodon as illustrated in the right table for the bicodon AAGAAG. In this particular case, the probability of obtaining such set of values (Fisher’s exact test) is around 5.3 × 10^−93^, consequently, we can reject the null hypothesis that this bicodon is equally likely to be in low and high PA samples. The formula to compute the p-value from the 2 × 2 contingency table is given in the bottom panel. [file peerj-05-3081-s012.pdf]

bicodon	Low PA	High PA
AAAAAA	460	132
AAAAAG	389	198
AAGAAA	494	401
AAGAAG	276	766

⇒

bicodon	Low PA	High PA
AAGAAG	276	766
others	1343	731

	Low PA	High PA
bicodon	a	b
others	c	d

$$p\text{-value} = \frac{(a+b)!\,(c+d)!(a+c)!(b+d)!}{(a+b+c+d)!\;a!\;c!\;b!\;d!}$$
