# Supplementary material for: Differential bicodon usage in lowly and highly abundant proteins
Source: PeerJ. 2017 Mar 9;5:e3081. doi: 10.7717/peerj.3081 (PMC5346287; doi:10.7717/peerj.3081)
Supplement: Supplemental Information 14 — Cell color is determined by the pause propensity score associated to each bicodon. The matrix of 3,904 (bicodons) × 9 (organisms) were grouped according to the pause propensity score similarity using average linkage clustering over the nine organisms. Then, for a better visualization, we display the pause propensity scores of each organism in a 61 × 64 grid, preserving the position of bicodons on the grid across the organisms. [file peerj-05-3081-s014.pdf]

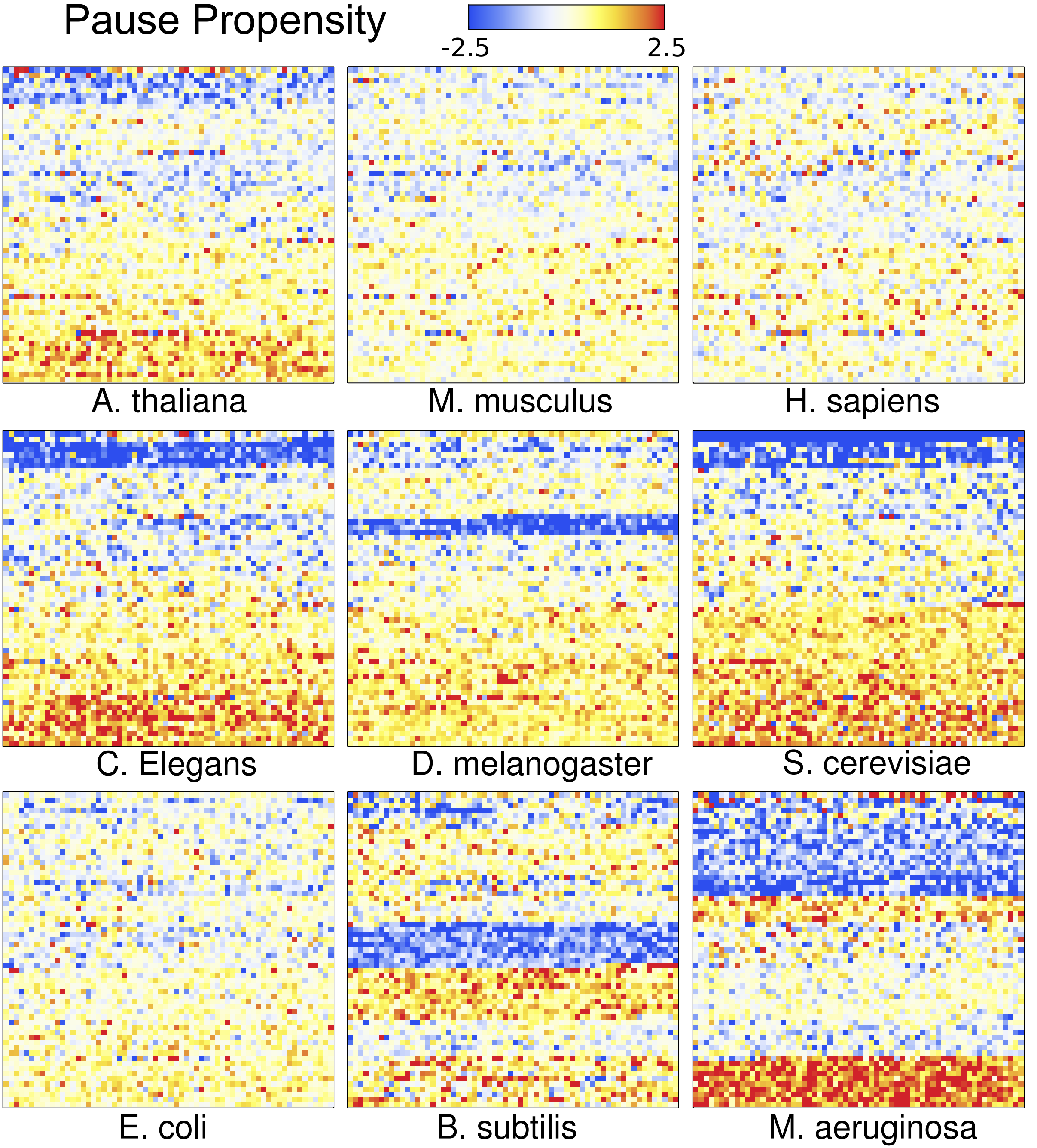

Pause Propensity
-2.5
2.5
A. thaliana
M. musculus
H. sapiens
C. Elegans
D. melanogaster
S. cerevisiae
E. coli
B. subtilis
M. aeruginosa
