# Supplementary material for: Differential bicodon usage in lowly and highly abundant proteins
Source: PeerJ. 2017 Mar 9;5:e3081. doi: 10.7717/peerj.3081 (PMC5346287; doi:10.7717/peerj.3081)
Supplement: Supplemental Information 15 — Cell color is determined by the quantity −Slog10(p-value), where p-value is provided by the Fisher’s exact test of the associated bicodon, S takes the values +1 or −1 when the bicodon has preference for sequences with low or with high PA. Thus, red cells indicate bicodons with clear preference for sequence associated to low PA, while blue cells indicate bicodons with high PA preference. [file peerj-05-3081-s015.pdf]

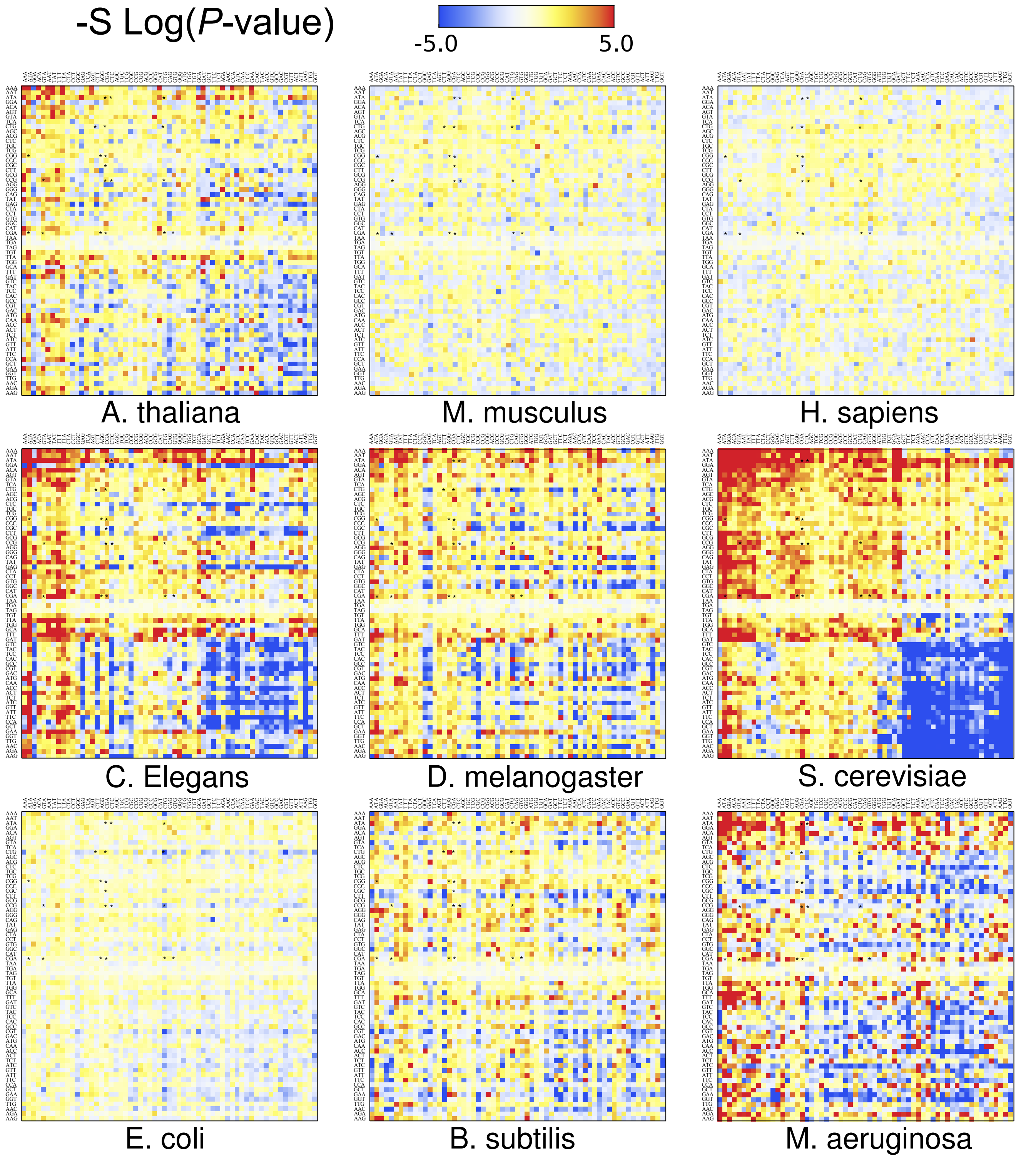

-S Log(P-value)
-5.0
5.0
A. thaliana
M. musculus
H. sapiens
C. Elegans
D. melanogaster
S. cerevisiae
E. coli
B. subtilis
M. aeruginosa
